# Induction Treatment for HIV-Associated Cryptococcal Meningitis: Where Have We Been and Where Are We Going?

**DOI:** 10.3390/microorganisms13040847

**Published:** 2025-04-08

**Authors:** Dominique Milsap, Madison Okuno, Enos Kigozi, Timothy Mugabi, Ssekindi Faizo, Aleksandra Bajer, Jane Gakuru, Nathan C. Bahr

**Affiliations:** 1 Department of Medicine, University of Minnesota, Minneapolis, MN 55455, USA; milsa004@umn.edu (D.M.); okuno004@umn.edu (M.O.); 2 Infectious Diseases Institute, Makerere University, Kampala, Uganda; kigozi.enos@gmail.com (E.K.); tmugabi@idi.co.ug (T.M.); fssekindi@idi.co.ug (S.F.); janegakuru18@gmail.com (J.G.); 3 Clinical Epidemiology Unit, School of Medicine, College of Health Sciences, Makerere University, Kampala, Uganda; 4 School of Medicine, University of Minnesota, Minneapolis, MN 55455, USA; bajer007@umn.edu; 5 Division of Infectious Diseases and International Medicine, Department of Medicine, University of Minnesota, 420 Delaware Street, SE, MMC 250, Minneapolis, MN 55455, USA

**Keywords:** cryptococcosis, cryptococcal meningitis, HIV/AIDS, antifungal medications, *Cryptococcus neoformans*

## Abstract

Cryptococcal meningitis remains a leading cause of morbidity and mortality among individuals with HIV/AIDS, particularly in resource-limited settings. Treatment begins with induction therapy followed by consolidation and maintenance. Evidence related to induction therapy has evolved significantly over the past decade. Current treatment relies primarily on three antifungal agents: amphotericin B, flucytosine, and fluconazole, each with distinct mechanisms of action and limitations. The World Health Organization’s 2022 guidelines for induction therapy recommend a single high dose of liposomal amphotericin B combined with 14 days of flucytosine and fluconazole. The 2010 IDSA guidelines for induction therapy recommend amphotericin B deoxycholate and flucytosine for two weeks. The U.S. CDC/NIH/IDSA/HIVMA joint guidelines and the ECCM/ISHAM/ASM joint guidelines list both options, but the recommendation varies by setting resources (e.g., resource-limited vs. other). The newer treatment approaches (single high-dose liposomal amphotericin B) that are supported by trials such as AMBITION-cryptococcal meningitis have limited adoption in high-resource settings, with recent studies showing that only 14% of North American infectious disease providers have utilized the regimen. Adjunctive medications, such as dexamethasone, tamoxifen, and sertraline, have proven ineffective or harmful in clinical trials. This review underscores the ongoing challenges in cryptococcal meningitis treatment and the need for continued research to improve patient outcomes, tracing the evolution from past monotherapy approaches to current combination strategies while exploring future directions.

## 1. Introduction

*Cryptococcus* is a yeast-like encapsulated fungus, with *Cryptococcus neoformans* and *Cryptococcus gattii* being the main pathogenic species complexes [1]. The fungus thrives primarily in soil containing bird droppings, and the *C. gattii* species complex is found worldwide and is reported frequently in the USA (Oregon, Washington, and California), Canada (British Columbia), Australia, Brazil, Colombia, and sub-Saharan Africa [2]. Transmission occurs through the inhalation of airborne fungal spores and desiccated yeasts. Once in the lungs, it can disseminate to other organs with a particular tropism for the central nervous system, causing meningitis. The polysaccharide capsule serves as a key virulence factor, enabling the evasion of the host immune responses. While immunocompetent individuals typically clear the *C. neoformans*, it poses a significant threat to immunocompromised patients. There is more variability among species within the *C. gattii* complex in terms of host immune response (e.g., some species more frequently affect those with or without compromised immune systems).

According to UNAIDS, approximately 38.6 million people worldwide were living with HIV in 2023 [3]. In patients with CD4 counts of <200 cells/µL, global cryptococcal antigenemia prevalence was estimated at 179,000 cases annually in 2020, with the highest burden of cryptococcal infections being in Sub-Saharan Africa and the Asia-Pacific region. Globally, 152,000 cases of cryptococcal meningitis occur annually, leading to 112,000 deaths (19% of AIDS-related deaths). Cryptococcal meningitis is the most common cause of adult meningitis among patients living with HIV, with a mortality of ~21–60%; however, fatality reaches 100% if untreated [4].

There have been significant changes over the past four decades to cryptococcal meningitis treatment, particularly with regards to induction therapy. Despite these advances, management remains challenging due to limited antifungal options and the need to balance tolerability with efficacy. The World Health Organization (WHO) 2022 guidelines [5], U.S. CDC/NIH/IDSA/HIVMA joint 2024 [6], and the European Confederation of Medical Mycology (ECMM)/International Society for Human and Animal Mycology (ISHAM)/American Society of Microbiology (ASM) 2024 guidelines [7] recommend induction therapy for patients with HIV with a single high dose (10 mg/kg) of liposomal amphotericin B combined with 14 days of flucytosine (100 mg/kg per day divided into four doses per day) and fluconazole (1200 mg daily), followed by fluconazole (800 mg daily) for eight weeks. The 2010 IDSA guidelines for induction therapy recommend amphotericin B deoxycholate (0.7–1.0 mg/kg per day) plus flucytosine (100 mg/kg per day) for two weeks [8]. The ECCM/ISHAM/ASM guidelines have subsequently been endorsed by the Infectious Diseases Society of America (IDSA).

However, there is variation in which setting the single high-dose approach is recommended for. The U.S. CDC/NIH/IDSA/HIVMA joint guidelines 2024 and the ECCM/ISHAM/ASM 2024 guidelines recommend daily amphotericin b-based induction therapy for fourteen days in high-resource settings for persons with HIV. In low-resource settings, they recommend single dose liposomal amphotericin b-based induction therapy.

Some guidelines recommend different induction regimens for persons without HIV. These nuances will not be discussed in detail in this review but are referenced in Table 1.

Understanding the evidence behind evolving treatment guidelines remains crucial for the optimal management of cryptococcal meningitis in both resource-rich and resource-limited settings.

## 2. Non-Standard or Previously Used Regimens

### 2.1. Fluconazole Monotherapy

Fluconazole inhibits ergosterol synthesis, a crucial component of fungal cell membranes [9]. Monotherapy with fluconazole is used for the treatment of cryptococcal meningitis in resource-limited settings where preferred options are not available due to its affordability, oral administration, and ease of use when compared to other regimens. Fluconazole has good cerebrospinal fluid penetration (CSF) and is generally well tolerated [9]. While there is evidence of clinical response and CSF sterilization with fluconazole, the widespread use of fluconazole monotherapy for cryptococcal meningitis in resource-limited settings has consistently demonstrated poorer clinical outcomes compared to amphotericin b-based therapies. Studies evaluating low-dose fluconazole (200–400 mg) demonstrate high mortality rates [10,11,12]. Research conducted in Malawi revealed that, even with high doses of fluconazole (800–1200 mg daily), mortality rates at 10 weeks reached 55–58%, despite higher doses being associated with greater rates of fungal clearance [13,14]. Similar findings have been reported in Uganda and South Africa [10,15,16].

The suboptimal outcomes associated with fluconazole are due to its fungistatic nature, which inhibits but does not kill *Cryptococcus neoformans*, resulting in slower clearance from CSF when compared with other treatments. Additionally, the development of drug resistance is a growing concern. Bicanic et al. identified that, among cases of cryptococcal meningitis relapse, 76% were associated with isolates, showing reduced susceptibility to fluconazole [10]. The fungistatic nature of fluconazole necessitates prolonged treatment durations, resulting in extended exposure and the development of resistant fungal organisms [17,18].

The current World Health Organization guidelines recommend fluconazole monotherapy only when amphotericin B is unavailable. In that case, fluconazole combined with flucytosine is preferred to fluconazole monotherapy [5,18]. We agree that fluconazole monotherapy should not be used unless none of the preferred options are available.

### 2.2. Amphotericin Monotherapy

Amphotericin B, first isolated from *Streptococcus* in 1955, belongs to the class of antifungals known as polyenes, which bind to ergosterol in the cell membrane, increasing membrane permeability and leading to cell death. This potent antifungal agent demonstrates fungicidal activity and excellent blood–brain barrier penetration [19]. Yet, amphotericin b monotherapy is suboptimal for cryptococcal meningitis compared to combination therapy regimens. In addition, inadequate access to amphotericin B products and their toxicity profiles limits its use. Clinical trials have consistently demonstrated that monotherapy leads to slower fungal clearance from CSF and higher mortality rates compared to combination approaches [20,21,22]. The toxicity profile of amphotericin B medications, particularly amphotericin B deoxycholate, presents significant risks. Prolonged amphotericin B use predictably leads to anemia, nephrotoxicity, and electrolyte imbalances, which can be fatal [10,23]. These adverse reactions, coupled with the need for intravenous administration and intensive monitoring, make extended amphotericin B therapy particularly problematic in resource-constrained settings where frequent and rapid laboratory monitoring can be difficult to accomplish. Generally, liposomal formulations are less toxic than amphotericin B deoxycholate, but they do still have significant toxicities. While amphotericin remains crucial in cryptococcal meningitis management, its role has transitioned from monotherapy to a key component of more effective and tolerable multi-drug regimens.

### 2.3. Amphotericin Plus Fluconazole

The combination of amphotericin and fluconazole would be ideal in some ways given the widespread availability of fluconazole compared to flucytosine. Although initial studies appeared promising, the combination of amphotericin B and fluconazole is now clearly understood to be only an alternative therapy for cryptococcal meningitis.

A 2004 study conducted in Thailand showed that amphotericin B deoxycholate (0.7 mg/kg/day) plus fluconazole (400 mg per day) did not show improved rates of CSF fungal clearance compared to amphotericin B deoxycholate alone [20]. A 2013 randomized controlled trial included 299 participants among three arms: (1) amphotericin deoxycholate (1 mg/kg/day) for 4 weeks, (2) amphotericin deoxycholate (1 mg/kg/day) and flucytosine (100 mg/kg/day) for 2 weeks, and (3) amphotericin deoxycholate (1 mg/kg/day) and fluconazole (400 mg twice daily) for 2 weeks [21]. This study found similar 10-week mortality rates comparing the combination arms (flucytosine vs. fluconazole). However, the fluconazole arm showed no significant mortality benefit compared to the amphotericin B deoxycholate monotherapy arm, while the flucytosine arm did show benefit compared to amphotericin B deoxycholate monotherapy at six months. In all, this hypothesis driving trial showed the need for a large, definitive, phase III study for these regimens.

The subsequent Antifungal Combinations for Treatment of Cryptococcal Meningitis in Africa (ACTA) trial showed definitively that flucytosine outperformed fluconazole when either agent was combined with amphotericin B deoxycholate, and this is discussed in detail below [18].

## 3. The ACTA Trial and Amphotericin Plus Flucytosine

The ACTA trial was an open-label, phase III, randomized, noninferiority, multicenter trial that enrolled a total of 721 adults with HIV and cryptococcal meningitis [18]. Participants were assigned to one of the following three arms:

1. An oral regimen (fluconazole [1200 mg per day] plus flucytosine [100 mg per kilogram of body weight per day] for 2 weeks).
2. One week of amphotericin B deoxycholate combination therapy (1 mg per kilogram per day).
3. Two weeks of amphotericin B deoxycholate combination therapy (1 mg per kilogram per day).

Each patient assigned to receive amphotericin B deoxycholate was also randomly assigned to receive fluconazole or flucytosine as a partner drug. Of note, flucytosine is a pyrimidine analog that is selectively taken up by fungal cells and disrupts fungal DNA and RNA synthesis. After induction treatment, all patients received fluconazole consolidation therapy and were followed for 10 weeks. The ACTA trial results showed that mortality rates in the oral regimen, one-week amphotericin B, and two-week amphotericin B groups were 18.2% (41 of 225), 21.9% (49 of 224), and 21.4% (49 of 229), respectively, at 2 weeks and 35.1% (79 of 225), 36.2% (81 of 224), and 39.7% (91 of 229), respectively, at 10 weeks.

As a partner drug with amphotericin B deoxycholate, flucytosine was superior to fluconazole (71 deaths [31.1%] vs. 101 deaths [45.0%]; hazard ratio for death at 10 weeks, 0.62; 95% confidence interval [CI], 0.45 to 0.84; *p* = 0.002). One week of amphotericin B deoxycholate plus flucytosine was associated with the lowest 10-week mortality rate (24.2%; 95% CI, 16.2 to 32.1). Adverse events, such as severe anemia, were more frequent with two weeks than with one week of amphotericin B deoxycholate or with the oral regimen. In summary, of the five regimens studied, one week of amphotericin B deoxycholate plus flucytosine was the most effective therapy, but outcomes were reasonable with an all-oral regimen of flucytosine plus fluconazole. Thus, if amphotericin-based therapy is not available, fluconazole plus flucytosine would be preferable to fluconazole alone, assuming that flucytosine is available.

While amphotericin B deoxycholate is more commonly available in low-resource settings, its more severe toxicity profile compared to liposomal amphotericin B is problematic. Thus, several studies have explored the use of daily liposomal amphotericin B (given at 3–5 mg/kg/day) in conjunction with flucytosine with the hope of better outcomes due to its good toxicity profile [24]. This has been adopted in high-resource settings.

Although it has become clear that flucytosine is a crucial component of our current best regimens, access has remained an issue in most low-resource settings outside of clinical trials (though with some improvement due to CDC/Unitaid/WHO efforts to improve access). Notably, flucytosine is dosed four times daily, which is also problematic. There is an ongoing phase II trial in Uganda assessing the efficacy of reduced dose and frequency of flucytosine in treatment of cryptococcal meningitis among adult patients with HIV [25].

## 4. AMBITION-cm

As noted above, ACTA clearly established amphotericin B plus flucytosine as the standard of care for induction therapy for cryptococcal meningitis. Yet, there remains significant potential to improve outcomes in cryptococcal meningitis.

The AMBisome Therapy Induction OptimizatioN (AMBITION-cm) trial, a multiple-site phase III randomized controlled trial, was conducted in Botswana, Malawi, South Africa, Uganda, and Zimbabwe from 2018 to 2021 [26,27]. A total of 844 participants were randomized to receive either a single high-dose (10 mg/kg) intravenous liposomal amphotericin B with 14 days of oral fluconazole (1200 mg/day) and oral flucytosine (100 mg/kg/day) in four divided doses, or the control group in which participants received one week of intravenous amphotericin B deoxycholate at 1 mg/kg/day with oral flucytosine at 100 mg/kg/day in four divided doses followed by one week of fluconazole at 1200 mg/day [27]. Single high-dose liposomal amphotericin B achieved non-inferiority compared to the control group. The experimental group had fewer adverse events and a 10-week mortality rate of 24.8% (95% CI 20.7–29.3%) compared to 28.7% (95%CI 24.4–33.4%) in the control group. These results led to changes in the 2022 World Health Organization guidelines to adopt the AMBITION-cm trial regimen as the first-line regimen for Human Immunodeficiency Virus-associated cryptococcal meningitis [5].

The current standard of care regimens are outlined in Table 1.

## 5. Adoption of ACTA/AMBITION-cm in Sub-Saharan Africa and Globally

The adoption of high-quality clinical trials is typically not immediate. The current practices of infectious disease clinicians in North America for the treatment of cryptococcal meningitis were recently surveyed via the Emerging Infections Network and the Mycoses Study Group Education and Research Consortium [28]. Their aim was to better understand treatment patterns for cryptococcal meningitis, including the adoption of the AMBITION-cm trial regimen. The survey revealed that 14% of providers who treated patients with cryptococcal meningitis used the single high-dose liposomal amphotericin B-based regimen. Among those who used the single high-dose liposomal amphotericin B regimen, 45% had used the regimen only among people with HIV, 17% used it in patients without HIV but with other immune-compromising conditions, and 38% used the regimen in both persons with and without HIV. Of the 86% of respondents who did not use the regimen, 16% said that they were unaware of the AMBITION-cm trial, and the remaining 84% were aware but chose not to use it. Perceived barriers to use it included uncertainty about efficacy in persons without HIV, uncertainty about the applicability of trials performed in low-resource settings compared to high-resource settings, and the lack of endorsement of this regimen in IDSA guidelines (which have not been updated since 2010 at the time of the survey—IDSA recently endorsed the ECCM/ISHAM/ASM guidelines).

In response to hypothetical clinical case scenarios about which treatment regimen respondents would use, 12% of providers stated that they would use the single high-dose liposomal amphotericin B regimen for a patient with HIV, versus 6% who said they would use the regimen in a person who had undergone a liver transplant, and 7% who would use it for a patient with cirrhosis. Most respondents (80%) selected the 2-week regimen recommended in the 2010 IDSA guidelines versus 12% who selected the AMBITION-cm regimen as their preferred regimen.

## 6. Possible Future Regimens

Suboptimal outcomes from current induction therapy, combined with the inadequate availability of flucytosine and liposomal amphotericin B in many high-burden, resource-limited settings, necessitate the rapid study of alternative regimens. Beyond modifications to existing therapies, as described in the ACTA and AMBITION-cm trials, other strategies include developing novel agents and repurposing existing drugs and non-pharmaceutical options. These possibilities are outlined in Table 2 and are described in more detail below.

### 6.1. Novel Agents

A novel lipid nanocrystal (LNC), oral amphotericin B, has been developed as an alternative to intravenous amphotericin B [29]. The LNC is engulfed by target cells (e.g., macrophages) and transported to infection sites. Its structure protects against degradation in harsh environments (e.g., acidic stomach pH), while enabling targeted intracellular delivery into macrophages and reticuloendothelial cells. Intracellularly, low calcium levels trigger the nanocrystals to release the drug inside the cell [30]. Orally administered LNC amphotericin showed in vitro activity against *Cryptococcus* and appeared to be synergistic with flucytosine [45]. A phase I trial reported good tolerability and safety of LNC amphotericin [31]. This led to a phase II randomized clinical (EnACT) trial, which compared LNC amphotericin with randomized controls receiving IV liposomal amphotericin B 3 mg/kg/d or IV amphotericin B deoxycholate 1 mg/kg/d. The results showed similar efficacy, CSF fungal activity, survival rates, and reduced toxicity compared to IV amphotericin B [30]. Whether this compound will undergo further study is uncertain.

Other promising new therapeutic agents include AP001 and its analogs (Fosmanogepix and APX2039), Mycograb, and oteseconazole (similar compounds originally developed by Viamet Pharmaceuticals). Fosmanogepix and APX2039 target the fungal enzyme Gwt1, disrupting glycosylphosphatidylinositol anchor synthesis, thereby affecting fungal growth [46]. APX2039 demonstrated activity against *Cryptococcus neoformans* in vitro and in various mammalian models of *cryptococcosis* [32]. *Fosmanogepix* has shown effectiveness in vitro against *C. neoformans*, and *C. gattii* and exhibited synergistic effects with fluconazole and liposomal amphotericin B [34,35]. In murine studies, fosmanogepix reduced cryptococcal fungal burden in the lungs and brain, and initial human trials have shown favorable side effect profiles [32,35]. Further randomized controlled trials are necessary to fully evaluate these agents.

Mycograb, a recombinant human antibody targeting heat shock protein 90 (Hsp90), was initially developed as an anticancer agent. Hsp90 is required for fungal cellular homeostasis; by inhibiting Hsp90, Mycograb has demonstrated in vitro activity against *Cryptococcus* [36,37]. Acting synergistically with amphotericin, mycograb has garnered interest, and phase II studies were planned [47,48]. However, all Hsp90 inhibitors developed to date have proven too immunosuppressive for antifungal use. Recent research indicates that variations in protein flexibility may enable the selective inhibition of fungal versus human Hsp90 isoforms, suggesting potential for this class of drugs [39].

There is a pressing need for new agents with greater specificity for fungal CYP51, a protein crucial for ergosterol synthesis. VT-1161 (oteseconazole), VT-1129 (quilseconazole), and VT-1598 inhibit CYP51 and block ergosterol biosynthesis in fungal cell membranes, exhibiting high potency against *Cryptococcus* species in vitro [40]. In a murine model of cryptococcal meningitis, these agents improved survival rates and reduced fungal burden [41]. Further studies assessing human safety and efficacy are warranted.

### 6.2. Repurposed Drugs

The challenges associated with the development of new antifungal agents have spurred an increasing interest in the repurposing of existing, safe, and affordable drugs.

#### 6.2.1. Dexamethasone

Dexamethasone has been shown to reduce mortality in human tuberculosis meningitis and showed promise in mouse models of *Cryptococcus gattii* infection [49]. This led to a double-blind, placebo-controlled trial assessing dexamethasone’s effects on mortality when added to standard treatment for cryptococcal meningitis [50]. This trial aimed to enroll 880 patients, but it was halted after enrolling 451 patients due to a higher risk of death and disability in the dexamethasone group compared to the placebo group at 10 weeks and 6 months. At 10 weeks, mortality rates were 47% for those on dexamethasone versus 41% for the placebo group; by six months, these figures rose to 57% and 49%, respectively. Additionally, patients receiving dexamethasone experienced fewer favorable outcomes (13% vs. 25%) and had slower fungal clearance from CSF.

#### 6.2.2. Tamoxifen

Tamoxifen, a well-established drug for breast cancer, has shown anti-cryptococcal activity when combined with other antifungals in mouse models [51]. A randomized controlled trial evaluated tamoxifen’s efficacy as an adjunct to amphotericin B and fluconazole in 50 patients [43]. The primary outcome measured was early fungicidal activity (EFA), defined as the reduction of culturable *Cryptococcus* in CSF during the initial two weeks. The results revealed no significant difference in EFA between the tamoxifen group and the control (−0.48 vs. −0.49 log10 CFU/mL/day). Mortality rates were comparable, with seven deaths in the tamoxifen group and eight in the control group by ten weeks. Patients receiving tamoxifen also had a lower percentage of favorable outcomes (9% vs. 36%). Moreover, QTc prolongation occurred in eight patients on tamoxifen. The overall findings did not support tamoxifen as a treatment for cryptococcal meningitis.

#### 6.2.3. Sertraline

Sertraline, a commonly prescribed antidepressant, has demonstrated anti-cryptococcal activity and synergy with fluconazole in vitro [52]. A dose-finding pilot study in Uganda suggested improved EFA with sertraline, prompting a larger phase III randomized placebo-controlled trial involving 460 participants [42]. This trial aimed to assess the impact of sertraline on survival, EFA, and adverse events. Ultimately, it was stopped for futility; mortality was 52% in the sertraline group compared to 46% in the placebo group. EFA rates were similar between groups, and both had comparable incidences of serious adverse events. While sertraline showed some benefits in reducing depression scores, it did not demonstrate a survival advantage or enhanced fungal clearance compared to the standard treatment.

The trials of dexamethasone, tamoxifen, and sertraline as adjunctive therapies for cryptococcal meningitis illustrate the challenges of repurposing existing drugs for this condition. Despite these unsuccessful clinical trials, the pursuit of affordable and accessible adjunctive treatments remains attractive to improve the management of cryptococcal meningitis.

#### 6.2.4. Other Possible Candidates

Miltefosine, an agent primarily used for leishmaniasis treatment, has in vitro activity and is effective in murine models of disseminated cryptococcosis [53,54]. Additional drug candidates are undergoing pre-clinical evaluation; for example, in vitro screening has identified 43 compounds capable of inhibiting *C. neoformans* growth, including ciclopirox and auranofin, although their advancement to clinical trials remains uncertain [55]. Antiprotozoal agents, such as benzimidazoles and flubendazole, have also shown potential in reducing fungal burden in infected murine models [56].

## 7. Non-Pharmaceutical Options

Neurapheresis CSF filtration is an emerging technique that filters CSF to remove pathogens and inflammatory mediators [57]. Initially designed for patients with hemorrhagic stroke, it has shown promise in safely filtering CSF to remove blood and byproducts [58]. This technology offers a potential one-time method to rapidly sterilize CSF in cryptococcal meningitis, possibly reducing the need for prolonged antifungal therapy. It could help alleviate increased intracranial pressure and enhance antifungal treatment effectiveness by lowering the fungal load in CSF. Further study is needed to understand whether any of these potential benefits truly occur.

## 8. Conclusions

Evidence for the treatment of cryptococcal meningitis has changed significantly over the last decade. Multiple large, high-quality trials have been completed, and the standard of care has completely shifted in people with advanced HIV. The single high-dose liposomal amphotericin-based regimen studied in the AMBITION-cm trial is now the first line therapy in people with advanced HIV. Yet, adoption has not been rapid in many locations. Some have questioned whether these results apply to higher resource settings (we believe they do), and others question whether they can be applied to other populations where the disease and presentations may differ compared to persons with advanced HIV. Though adoption has not been universal, uptake has been strong in many locations, with some locations publishing real-world data that demonstrate outcomes consistent with those seen in the trial [26]. However, further data, including among individuals without HIV, are needed. Therefore, we encourage providers working with these populations to investigate this regimen further in these groups as well.

Progress in the study of HIV-associated cryptococcal meningitis is likely to continue, driven by multiple dedicated and scientifically rigorous groups committed to advancing research, assuming similar commitments from funders. These groups are actively conducting additional studies, including a platform trial, to further expand our knowledge and improve outcomes [59]. Thus, decisions about how trials conducted in persons with HIV relate to persons without HIV will remain a major issue in cryptococcal meningitis.

## Figures and Tables

**Table 1:** Currently utilized induction treatment regimens for cryptococcal meningitis.

Regimen	WHO Guideline Endorsed (2022) [5]	IDSA Guideline Endorsed (2010) [8]	U.S. CDC/NIH/IDSA/HIVMA Guideline Endorsed (2024) [6]	ECCM/ISHAM/ASM Guideline Endorsed (2024) [7]
A single high dose (10 mg/kg) of liposomal AmB with 2 weeks of flucytosine (100 mg/kg per day divided into four doses per day) and fluconazole (1200 mg daily)	Primary Regimen ^a^	No	Primary Regimen ^a,k^	Primary Regimen ^a,k^
1 week of AmBd (1 mg/kg per day) and flucytosine (100 mg/kg per day, divided into four doses per day) followed by 1 week of fluconazole (1200 mg daily)	Alternative Regimen ^a^	No	Alternative Regimen ^a^	Alternative Regimen ^a^
2 weeks of liposomal AmB (3–4 mg/kg per day) plus flucytosine (100 mg/kg per day) ^d^	No	Primary Regimen ^a,b^	Primary Regimen ^a,j^	Primary Regimen ^a,b,c,j^
2 weeks of AmBd (0.7–1.0 mg/kg per day) plus flucytosine (100 mg/kg per day)	No	Primary regimen ^a^	No	Alternative Regimen ^b,c^
2 weeks of ABLC (5 mg/kg per day, with renal function concerns) plus flucytosine (100 mg/kg per day)	No	Primary Regimen ^a,b^	No	Alternative Regimen ^a,b,c^
2 weeks of fluconazole (1200 mg daily) + flucytosine (100 mg/kg per day, divided into four doses per day)	Alternative Regimen ^a^	No	No	Alternative Regimen ^a,b,c^
2 weeks of liposomal AmB (3–4 mg/kg per day) + fluconazole (1200 mg daily)	Alternative Regimen ^a^	No	No	Alternative Regimen ^a^
2 weeks of AmBd (1 mg/kg per day) + fluconazole (1200 mg daily)	Alternative Regimen ^a^	No	No	Alternative Regimen ^a^
2 weeks of ABLC (5 mg/kg per day) plus fluconazole (800–1200 mg per day)	No	No	No	Alternative Regimen ^a^
4–6 weeks of AmBd (0.7–1.0 mg/kg per day) or liposomal AmB (3–4 mg/kg per day) or ABLC (5 mg/kg per day, for flucytosine-intolerant patients)	No	Primary Regimen ^a^	No	
≥4 weeks of AmBd (0.7–1.0 mg/kg per day) plus flucytosine (100 mg/kg per day) ^f^	No	Primary regimen ^c^	No	
≥6 weeks of AmBd (0.7–1.0 mg/kg per day) ^f,g^	No	Primary regimen ^c^	No	
≥4 weeks of liposomal AmB (3–4 mg/kg per day) or ABLC (5 mg/kg per day) combined with flucytosine, if possible ^f,h^	No	Primary regimen ^c^	No	
2 weeks of AmBd (0.7 mg/kg per day) plus flucytosine (100 mg/kg per day) ^i^	No	Primary regimen ^c^	No	Alternative Regimen ^a^
4–6 weeks of liposomal AmB (6 mg/kg per day) or ABLC (5 mg/kg per day)	No	Alternative Regimen) ^b^	No	
4–6 weeks of AmBd (0.7 mg/kg per day) ^e^	No	Alternative Regimen) ^b^	No	
2 weeks of AmBd (0.7 mg/kg per day IV) plus fluconazole (800 mg per day orally), followed by a minimum of 8 weeks of fluconazole (800 mg per day orally)	No	Alternative Regimen ^a^	No	
6 weeks of fluconazole (≥800 mg per day orally; 1200 mg per day is favored) plus flucytosine (100 mg/kg per day orally)	No	Alternative Regimen ^a^	No	
10–12 weeks of fluconazole (800–2000 mg per day orally)	No	Alternative Regimen ^a^	No	
10–12 weeks of itraconazole (200 mg twice per day orally)	No	Alternative Regimen ^a^	No	
2 weeks of fluconazole (800–1200 mg daily)				Alternative Regimen ^a^

NOTE. AmB, Amphotericin B; AmBd, Amphotericin B deoxycholate; ABLC, Amphotericin B lipid complex. ^a^ Antifungal treatment recommendation in HIV-infected individuals. ^b^ Antifungal treatment recommendation in transplant individuals. ^c^ Antifungal treatment recommendation in non-HIV-infected, non-transplant individuals. ^d^ Immunosuppressive management may require sequential or stepwise reductions. ^e^ Many transplant recipients have been successfully treated with AmBd; however, issues of renal dysfunction with calcineurin inhibitors are important, and the effective dose is imprecise. ^f^ Four weeks are reserved for patients with meningitis who have no neurological complications, who have no significant underlying diseases or immunosuppression, and for whom the cerebrospinal fluid culture performed at the end of 2 weeks of treatment does not yield viable yeasts; during the second 2 weeks, lipid formulations of AmB may be substituted for AmBd. ^g^ For flucytosine-intolerant patients. ^h^ For AmBd-intolerant patients. ^i^ For patients who have a low risk of therapeutic failure. Low risk is defined as an early diagnosis by history, no uncontrolled underlying condition or severe immunocompromised state, and an excellent clinical response to the initial 2-week antifungal combination course. ^j^ Preferred in high-income settings. ^k^ Recommended in low-income settings.

**Table 2:** Possible treatments of interest that require further study for cryptococcal meningitis.

Therapy	Mechanism of Action/Formulation Information	Existing Evidence	Current or Planned Activity
Oral amphotericin B [29]	Novel encochleated amphotericin B deoxycholate formulation has oral bioavailability and minimal toxicity due to targeted drug delivery into macrophages, where intracellular fungi reside [30]	In vitro activity against Cryptococcus; synergistic with flucytosine [30] Phase I trial reported good tolerability and safety [31] Phase II EnACT trial: Similar efficacy, CSF fungal activity, similar survival, and less toxicity than IV amphotericin [30]	Phase III trial on hold
APX2039 [32]	Gwt1 inhibitor	In vitro activity against *Cryptococcus neoformans* and various mammalian models of cryptococcosis	NA
Fosmanogepix (APX001)	Gwt1 inhibitor prevents the appropriate localization of cell wall mannoproteins, compromising cell wall integrity, biofilm and germ tube formation, and fungal growth [33]	Effective in vitro against various pathogenic fungi and exhibits synergistic effects with fluconazole [34] and liposomal amphotericin B [35] Reduced cryptococcal loads in the lungs and brain [34]	NA
Mycograb	Recombinant human antibody that specifically targets heat shock protein 90 (Hsp90) to disrupt normal cellular functions and compromise the structural integrity essential for fungal survival [36]	Hsp90 inhibition has demonstrated in vitro activity against Cryptococcus [37] All Hsp90 inhibitors developed to date have proven too immunosuppressive for antifungal use [38]	Variations in protein flexibility may enable the selective inhibition of fungal versus human Hsp90 isoforms [39]
VT-1161 (oteseconazole), VT-1129 (quilseconazole), and VT-1598	Inhibit CYP51 and block ergosterol biosynthesis in fungal cell membranes [40]	In a murine model of cryptococcal meningitis, these agents improved survival rates, reduced fungal burden, and demonstrated persistence in brain tissue [41]	NA
Sertraline [42]	Selective serotonin reuptake inhibitor antidepressant	Demonstrated in vitro fungicidal activity against *Cryptococcus neoformans* and synergy with fluconazole ASTRO-cryptococcal meningitis pilot study assessed optimal sertraline dosing for HIV-associated cryptococcal meningitis and appeared to improve the rate of CSF cryptococcal clearance when compared to historical controls Phase III trial evaluating the efficacy of amphotericin B and fluconazole, with or without sertraline 400 mg for two weeks, in the treatment of HIV-associated cryptococcal meningitis. Did not demonstrate reduction in mortality or CSF fungal clearance and was halted for futility	NA
Tamoxifen [43]	Selective estrogen receptor modulator that exhibits anti-cryptococcal effects and synergizes with amphotericin B and fluconazole in vitro	A phase II trial added tamoxifen (300 mg/day) to standard induction therapy for two weeks. No significant difference in CSF fungal clearance	NA
Miltefosine	An antileishmanial agent. Mechanism not well understood; inhibits the synthesis of phosphatidylcholine and also affects the parasite mitochondrion, inhibiting the cytochrome c oxidase [33]	In vitro and murine models of disseminated cryptococcosis have shown effectiveness [38]	NA
Antiprotozoal agents (i.e., Benzimidazoles) [44]	Disrupt microtubules through binding to the β-tubulin subunit	Potential in reducing fungal burden in infected murine models	NA
Neuraphresis CSF filtration [38]	Filters CSF to remove pathogens and inflammatory mediators. Offers a potential one-time method to rapidly sterilize CSF in cryptococcal meningitis	Potential one-time method to rapidly sterilize CSF in cryptococcal meningitis, possibly reducing the need for prolonged antifungal therapy May help alleviate increased intracranial pressure and enhance antifungal treatment effectiveness by lowering the fungal load in CSF	NA

NA: not applicable.
